# Real-world outcomes of nivolumab plus ipilimumab and pembrolizumab with platinum-based chemotherapy in advanced non-small cell lung cancer: a multicenter retrospective comparative study

**DOI:** 10.1007/s00262-023-03583-4

**Published:** 2024-01-04

**Authors:** Kinnosuke Matsumoto, Takayuki Shiroyama, Motohiro Tamiya, Toshiyuki Minami, Yuhei Kinehara, Akihiro Tamiya, Yasuhiko Suga, Tomoki Kuge, Masahide Mori, Hidekazu Suzuki, Satoshi Tobita, Kiyonobu Ueno, Yoshinobu Namba, Satoshi Tetsumoto, Toshie Niki, Osamu Morimura, Akio Osa, Kazumi Nishino, Izumi Nagatomo, Yoshito Takeda, Takashi Kijima, Atsushi Kumanogoh

**Affiliations:** 1 Department of Respiratory Medicine and Clinical Immunology, Graduate School of Medicine, Osaka University, 2- 2 Yamadaoka, Suita City, 565-0871 Osaka Japan; 2 Department of Respiratory Medicine, Osaka International Cancer Institute, Osaka, Japan; 3 Department of Respiratory Medicine and Hematology, Hyogo Medical University, Hyogo, Japan; 4 Department of Respiratory Medicine and Clinical Immunology, Nippon Life Hospital, Osaka, Japan; 5 Department of Internal Medicine, National Hospital Organization Kinki-Chuo Chest Medical Center, Osaka, Japan; 6 Department of Respiratory Medicine, Osaka Police Hospital, Osaka, Japan; 7 Department of Thoracic Oncology, National Hospital Organization Osaka Toneyama Medical Center, Osaka, Japan; 8 Department of Thoracic Oncology, Osaka Habikino Medical Center, Osaka, Japan; 9 Department of Respiratory Medicine, Osaka General Medical Center, Osaka, Japan; 10 Department of Respiratory Medicine, Takarazuka City Hospital, Hyogo, Japan; 11 Department of Respiratory Medicine and Clinical Immunology, Suita Municipal Hospital, Osaka, Japan; 12 Department of Respiratory Medicine, Nishinomiya Municipal Central Hospital, Hyogo, Japan; 13 Department of Respiratory Medicine, Toyonaka Municipal Hospital, Osaka, Japan; 14 Department of Respiratory Medicine, Kinki Central Hospital, Hyogo, Japan; 15 Department of Immunopathology, Immunology Frontier Research Center (iFReC), World Premier International (WPI), Osaka University, Osaka, Japan; 16 Integrated Frontier Research for Medical Science Division, Institute for Open and Transdisciplinary Research Initiatives (OTRI), Osaka University, Osaka, Japan; 17 Center for Infectious Diseases for Education and Research (CiDER), Osaka University, Suita, Osaka Japan; 18 Japan Agency for Medical Research and Development – Core Research for Evolutional Science and Technology (AMED–CREST), Osaka University, Osaka, Japan; 19 Center for Advanced Modalities and DDS (CAMaD), Osaka University, Osaka, Japan

**Keywords:** Nivolumab, Ipilimumab, Pembrolizumab, Non-small-cell Lung cancer, Propensity score, Real-world

## Abstract

**Introduction:**

Nivolumab plus ipilimumab with chemotherapy (NICT) and pembrolizumab with chemotherapy (PCT) are commonly used in patients with advanced non-small cell lung cancer (NSCLC). Compared with immune checkpoint inhibitor (ICI) monotherapy, ICI combination therapy can increase immune-related toxicity instead of prolonging survival. This study aimed to compare the efficacy and safety of NICT and PCT to decide on the favorable treatment.

**Methods:**

We conducted a multi-center retrospective cohort study on patients who underwent NICT or PCT between December 2018 and May 2022. Propensity score matching (PSM) was performed with the variables age, sex, smoking status, performance status, stage, histology, and programmed cell death ligand-1 (PD-L1). The Kaplan–Meier method was used to compare survival for the matched patients.

**Results:**

Six hundred consecutive patients were included. After PSM, 81 and 162 patients were enrolled in the NICT and PCT groups, respectively. The baseline characteristics were well-balanced. The median progression-free survival was equivalent (11.6 vs. 7.4 months; *P* = 0.582); however, the median overall survival (OS) was significantly longer in the NICT group than in the PCT group (26.0 vs. 16.8 months; *P* = 0.005). Furthermore, OS was better in PD-L1-negative patients who underwent NICT than in those who underwent PCT (26.0 vs. 16.8 months; *P* = 0.045). Safety profiles did not differ significantly in terms of severe adverse event and treatment-related death rates (*P* = 0.560, and 0.722, respectively).

**Conclusions:**

Real-world data suggests that NICT could be a favorable treatment option compared with PCT for patients with advanced NSCLC. Further follow-up is needed to determine the long-term prognostic benefit.

**Supplementary Information:**

The online version contains supplementary material available at 10.1007/s00262-023-03583-4.

## Introduction

Anti-programmed cell death-1 (PD-1), anti-programmed cell death ligand-1 (PD-L1), and anti-cytotoxic T-lymphocyte-associated protein 4 (CTLA-4) antibodies combined with platinum-based chemotherapy have drastically improved the survival outcomes of patients with advanced non-small cell lung cancer (NSCLC) [1–6]. Nivolumab plus ipilimumab with chemotherapy (NICT) and pembrolizumab with chemotherapy (PCT) were approved based on phase III randomized controlled trials (RCTs), including the CheckMate-9LA and KEYNOTE-189/407 studies [1–3]. They are among the most common first-line treatments for patients with advanced NSCLC with wild-type EGFR and ALK, as their efficacies have been demonstrated regardless of histology or PD-L1 expression levels.

Ipilimumab, an anti-CTLA-4 IgG1 monoclonal antibody, induces a durable immune response by directly binding to and activating T cells while reducing regulatory T cells. Generally, the addition of anti-CTLA-4 antibody to anti-PD-1 or PD-L1 antibody provides longer survival benefits but also increases the frequency of immune-related adverse events (AEs) [6, 7]. Two network meta-analyses indirectly compared data retrieved from the above-mentioned RCTs and reported no significant differences among patients with NSCLC without PD-L1 selection between NICT and PCT in overall survival (OS) and AEs ≥ grade 3 [8, 9]. A phase III clinical trial directly comparing NICT and PCT (the NIPPON study) was initiated in April 2021; however, the NICT group reported treatment-related deaths beyond the expected range, leading to premature termination of the trial before its outcomes were available [10, 11].

Ultimately, determining the appropriate treatment for treatment-naïve advanced NSCLC is difficult, given the lack of clinical studies on direct comparisons between NICT and PCT in terms of efficacy and safety. Moreover, RCTs are often highly selective and low-risk, yielding results that cannot be generalized to patient groups in real-world settings [12]. Therefore, it is plausible that real-world scenarios might entail more severe toxicity compared to that observed in the NIPPON study. This highlights the urgent need to elucidate and compare the efficacy and safety of NICT and PCT using real-world data in terms of treatment options. Thus, this study aimed to retrospectively evaluate the efficacy and safety of NICT and PCT in treatment-naïve patients with advanced NSCLC using propensity score matching (PSM) to reduce the impact of differences in baseline characteristics between treatment groups.

## Materials and methods

### Patient selection

Consecutive patients with histologically confirmed advanced or recurrent-stage NSCLC were registered through the electronic databases of 14 institutes in Japan: those who were treated with a first-line combination of nivolumab plus ipilimumab or pembrolizumab with platinum-based chemotherapy were included, and those with major EGFR gene mutation (exon 21 L858R or exon 19 deletion) mutations and ALK/ROS1 rearrangements were excluded. Patients for whom treatment was initiated between December 2018 and May 2022 were included, and the cutoff date for data collection was May 31, 2023.

### Study design

This was a multicenter retrospective cohort study. The patients were classified into two groups according to the first-line treatment type: the NICT group, including patients treated with nivolumab plus ipilimumab with platinum-based chemotherapy; and the PCT group, including patients treated with pembrolizumab with platinum-based chemotherapy.

Clinical data collected from medical records included age, sex, smoking status, Eastern Cooperative Oncology Group Performance Status (ECOG-PS), stage, driver gene mutation, histology, PD-L1 expression, previous thoracic radiotherapy, treatment outcomes, and AEs. Clinical responses were defined according to the Response Evaluation Criteria in Solid Tumors (RECIST) version 1.1 [13]. Time to treatment discontinuation (TTD) was defined as the period from the first-line treatment start date to the date of discontinuation for any cause. Progression-free survival (PFS) was defined as the period from the first-line treatment start date to the date of disease progression or death from any cause, and OS was determined from the first-line treatment start date to the date of death or last follow-up. The safety level was evaluated using the Common Terminology Criteria for Adverse Events, version 5.0 (CTCAE, ver5) based on AE incidence, treatment discontinuation, and treatment-related death (TRD) [14]. In this study, severe AEs (SAEs) were defined as AEs ≥ grade 3.

### Statistical analyses

The sample size was determined based on the number of patients who met the inclusion criteria. The primary endpoints were TTD, PFS, OS, and SAE profiles between the matched groups. The secondary endpoint was the OS in each subgroup. We used a 1:2 propensity score-nearest neighbor matched pair method with a caliper size of 0.2. Unbalanced baseline conditions between groups were controlled through PSM with covariate adjustments for age, sex, smoking status, ECOG-PS, stage, histology, and PD-L1 levels [1–3]. Comparisons between groups were performed using the Mann–Whitney U-test for continuous data and the chi-squared test for categorical data before and after PSM. Fisher’s exact test was used for analysis when the smallest expected value was < 5. Survival was assessed using the Kaplan–Meier method, with the log-rank test for comparison. The median follow-up duration was calculated using only patients without death events. The Cox proportional hazards regression model was used for the univariate analysis of TTD, PFS, and OS. Differences with two-sided *p* values < 0.05 were considered significant. Statistical analyses were performed using SPSS, version 28.0 (IBM, Armonk, NY, USA).

## Results

### Patient characteristics

Among 600 eligible patients, 83 (13.8%) and 517 (86.2%) were classified into the NICT and PCT groups, respectively (Fig. S1). Baseline demographic and clinical characteristics of the study patients are presented in Table 1. No significant differences were noted in age, sex, smoking history, ECOG-PS, stage, histology, or previous thoracic radiotherapy between the two groups; however, a large difference was noted in the proportion of PD-L1 tumor proportion score (TPS). After PSM, a total of 243 patients were enrolled in the study, with 81 and 162 patients in the NICT and PCT groups, respectively. Baseline characteristics, including the PD-L1 TPS, did not differ significantly between the matched groups.

**Table 1:** Patient characteristics before and after propensity score matching (PSM)

	All pts in the cohort (n = 600)			Pts adjusted after PSM (n = 243)		
	NICT (n = 83)	PCT (n = 517)	*P* value	NICT (n = 81)	PCT (n = 162)	*P* value
Median age (range)	69 [36–84]	69 [36–84]	0.196	69 [36–84]	68 [43–80]	0.820
< 65	31 (37.3)	161 (31.1)	0.257	29 (35.8)	59 (36.4)	1.000
≥ 65	52 (62.7)	356 (68.9)		52 (64.2)	103 (63.6)	
Sex, n (%)			0.255			0.714
Female	14 (16.9)	118 (22.8)		14 (17.3)	25 (15.4)	
Male	69 (83.1)	399 (77.2)		67 (82.7)	137 (84.6)	
Smoking, n (%)			0.133			1.000
Never	5 (6.0)	61 (11.8)		5 (6.2)	9 (5.6)	
Former/current	78 (94.0)	456 (88.2)		76 (93.8)	153 (94.4)	
ECOG-PS, n (%)			0.453			1.000
0/1	72 (86.7)	462 (89.4)		71 (87.7)	142 (87.7)	
2/3/4	11 (13.3)	55 (10.6)		10 (12.3)	20 (12.3)	
Stage, n (%)			0.890			0.877
IV	63 (75.9)	396 (76.6)		61 (75.3)	119 (73.5)	
Others	20 (24.1)	121 (23.4)		20 (24.7)	43 (26.5)	
III	10 (12.0)	40 (7.7)		10 (12.3)	13 (8.0)	
Recurrent after surgery	7 (8.4)	74 (14.3)		7 (8.6)	27 (16.7)	
Recurrent after radiotherapy	3 (3.6)	7 (1.4)		3 (3.7)	3 (1.9)	
Histology, n (%)			0.088			0.666
Adenocarcinoma	45 (54.2)	299 (57.8)		45 (55.6)	91 (56.2)	
Squamous cell	25 (30.1)	177 (34.2)		25 (30.9)	55 (34.0)	
Others	13 (15.7)	41 (7.9)		11 (13.6)	16 (9.9)	
PD-L1 TPS, n (%)			0.001			0.553
≥ 50%	10 (12.0)	157 (30.4)		10 (12.3)	24 (14.8)	
1–49%	32 (38.6)	162 (31.3)		31 (38.3)	55 (34.0)	
<1%	35 (42.2)	142 (27.5)		34 (42.0)	62 (38.3)	
unknown	6 (7.2)	56 (10.8)		6 (7.4)	21 (13.0)	

Abbreviations: NICT, nivolumab plus ipilimumab with chemotherapy; PCT, pembrolizumab with chemotherapy; ECOG-PS, Eastern Cooperative Oncology Group Performance Status; PD-L1 TPS, programmed cell death ligand-1 tumor proportion score; pts, patients

### Treatment effectiveness in all matched patients

**Fig. 1:**
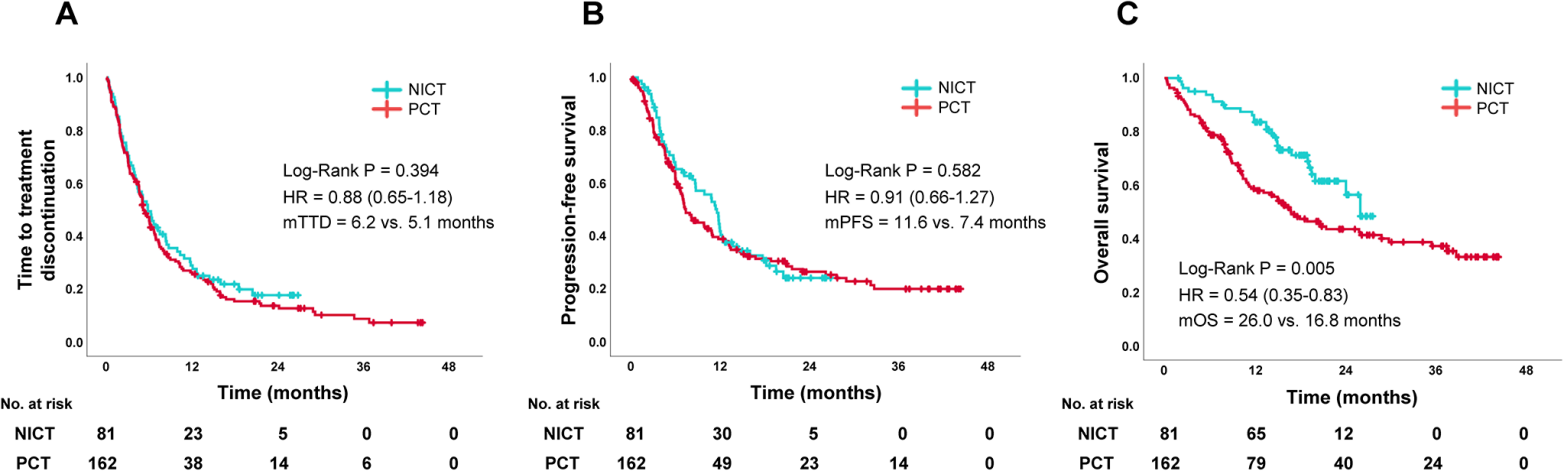
Kaplan–Meier survival curves of TTD (**A**), PFS (**B**), and OS (**C**) in the NICT and PCT groups after propensity score matching. Abbreviations: NICT, nivolumab plus ipilimumab with chemotherapy; PCT, pembrolizumab with chemotherapy; mTTD, median time to treatment discontinuation; mPFS, median progression-free survival; mOS, median overall survival; HR, hazard ratio

The median follow-up period was 18.5 (interquartile range, 14.6–22.1) and 20.9 (interquartile range, 9.8–37.5) months among patients treated with NICT and PCT, respectively (*P* = 0.291). The median TTD was 6.2 and 5.1 months [hazard ratio (HR): 0.88, 95% confidence interval (CI): 0.65–1.18, *P* = 0.394, Fig. 1A)] and the median PFS was 11.6 and 7.4 months (HR: 0.91, 95%CI: 0.66–1.27, *P* = 0.582, Fig. 1B) in the NICT and PCT groups, respectively. Moreover, the median OS was significantly longer in the NICT group than in the PCT group (26.0 vs. 16.8 months, HR: 0.54, 95%CI: 0.35–0.83, *P* = 0.005, Fig. 1C).

### Treatment effectiveness in each subgroup

**Fig. 2:**
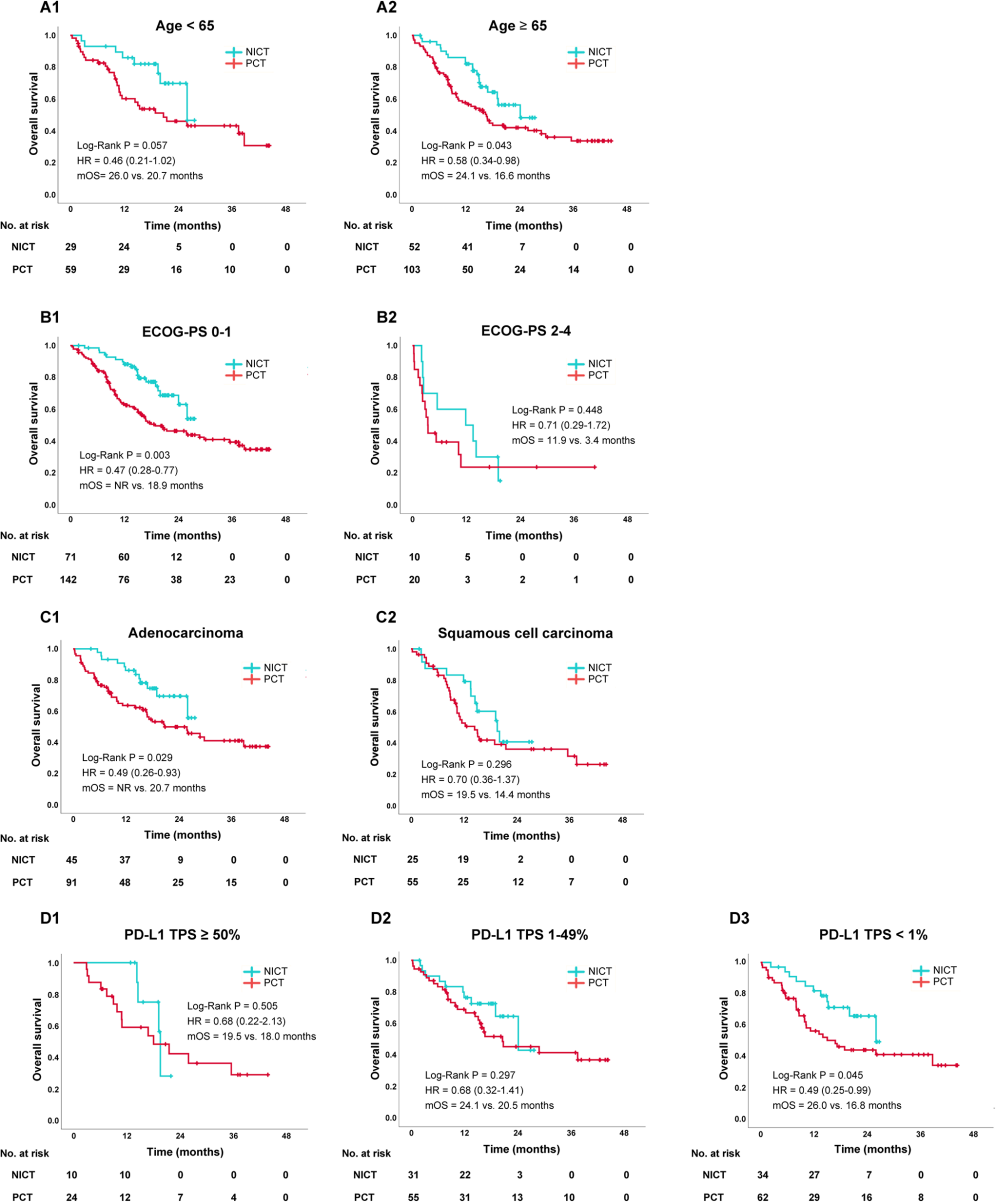
Subgroup analyses for OS in the NICT and PCT groups after a propensity score matching method according to age (**A1**: <65 years, **A2**: ≥65 years), ECOG-PS (**B1**: PS 0–1, **B2**: PS 2–4), histology (**C1**: adenocarcinoma, **C2**: squamous cell carcinoma), and PD-L1 TPS (**D1**: TPS ≥ 50%, **D2**: TPS 1–49%, **D3**: TPS < 1%). Abbreviations: NICT, nivolumab plus ipilimumab with chemotherapy; PCT, pembrolizumab with chemotherapy; mOS, median overall survival; HR, hazard ratio; NR, not reached; ECOG-PS, Eastern Cooperative Oncology Group Performance Status; PD-L1 TPS, programmed cell death ligand-1 tumor proportion score

We analyzed the OS, TTD and PFS in certain subgroups. A trend for longer OS was observed in patients who received NICT than in those who received PCT in both younger (26.0 vs. 20.7 months; HR, 0.46; 95% CI, 0.21–1.02; *P* = 0.057) and elderly (24.1 vs. 16.6 months; HR, 0.58; 95% CI, 0.34–0.98; *P* = 0.043) patients (Fig. 2A1 and A2), respectively. A similar trend of better OS in patients receiving NICT was observed in the ECOG-PS 0–1 group alone [not reached (NR) vs. 18.9 months; HR, 0.47; 95% CI, 0.28–0.77; *P* = 0.003)] (Fig. 2B1 and B2). Histologically, NICT was more significantly associated with longer OS than PCT for adenocarcinoma (NR vs. 20.7 months; HR, 0.49; 95% CI, 0.26–0.93; *P* = 0.029) but not for squamous cell carcinoma (19.5 vs. 14.4 months; HR, 0.70; 95% CI, 0.36–1.37; *P* = 0.30) (Fig. 2C1 and C2). When subgrouping based on PD-L1 expression levels, the OS was significantly longer in the NICT group than in the PCT group only in PD-L1 TPS-negative cases (26.0 vs. 16.8 months; HR, 0.49; 95% CI, 0.25–0.99; *P* = 0.045) (Fig. 2D1–3). In contrast, the TTD and PFS were almost equal between the NICT and PCT groups in all subgroup analyses (Fig. S2 and S3).

### Other treatment characteristics for matched patients

Approximately 60% of patients in both groups received platinum doublets that included pemetrexed, whereas the others received platinum doublets that included paclitaxel (*P* = 0.674) (Table S1). Approximately 50% of patients in both groups received only first-line treatment at the time of the analysis, and no significant differences were noted between the two groups (*P* = 0.102). For NICT and PCT, the objective response rates were 71.6% and 58.0% (*P* = 0.039), and the disease control rates were 87.7% and 87.0% (*P* = 0.892), respectively.

### Safety profiles

Table 2 summarizes the treatment-related SAEs within 12 months after initiation of combination therapy. In total, 28 (34.6%) and 50 (30.9%) patients in the NICT and PCT groups, respectively, experienced grade ≥ 3 SAEs (*P* = 0.560), and 15 (18.5%) and 31 (19.1%) patients discontinued treatment owing to SAEs, respectively (*P* = 0.908). Two (2.5%) and six (3.7%) patients died from treatment-related AEs (*P* = 0.722): one of pneumonitis and one of steroid-related infection in the NICT group and four of pneumonitis and two of colitis in the PCT group. Among the patients who developed SAEs, the most frequent event was pneumonitis (8.6% of patients); however, no significant difference was noted between the NICT and PCT groups (*P* = 0.333). Among all SAEs, skin and hepatobiliary toxicities and adrenal pituitary disorder occurred more frequently in the NICT group than in the PCT group, although the difference was not significant.

**Table 2:** Treatment-related severe adverse events (SAEs)^a^ within 12 months after initiation of combination therapy

	NICT (n = 81)	PCT (n = 162)	*P* value
Number of patients with SAEs, (%)	28 (34.6)	50 (30.9)	0.560
SAEs leading to discontinuation, n (%)	15 (18.5)	31 (19.1)	0.908
Treatment related death, n (%)^b^	2 (2.5)	6 (3.7)	0.722
SAEs occurring in ≥ 3 patients, n (%)			
Pneumonitis	5 (6.2)	16 (9.9)	0.333
Skin toxicity	6 (7.4)	3 (1.9)	0.064
Colitis	4 (4.9)	7 (4.3)	1.000
Hepatobiliary toxicity	6 (7.4)	3 (1.9)	0.064
Renal toxicity	2 (2.5)	3 (1.9)	1.000
Adrenal pituitary disorder	6 (7.4)	4 (2.5)	0.088
Febrile neutropenia	2 (2.5)	5 (3.1)	1.000
Others (details unknown)	1 (1.2)	7 (4.3)	

^a^ Severe adverse events indicate grade 3 or higher

^b^ Treatment related deaths include one pneumonitis case and one steroid-related infection in the NICT group and four pneumonitis cases and two colitis cases in the PCT group

Abbreviations: NICT, nivolumab plus ipilimumab with chemotherapy; PCT, pembrolizumab with chemotherapy

**Fig. 3:**
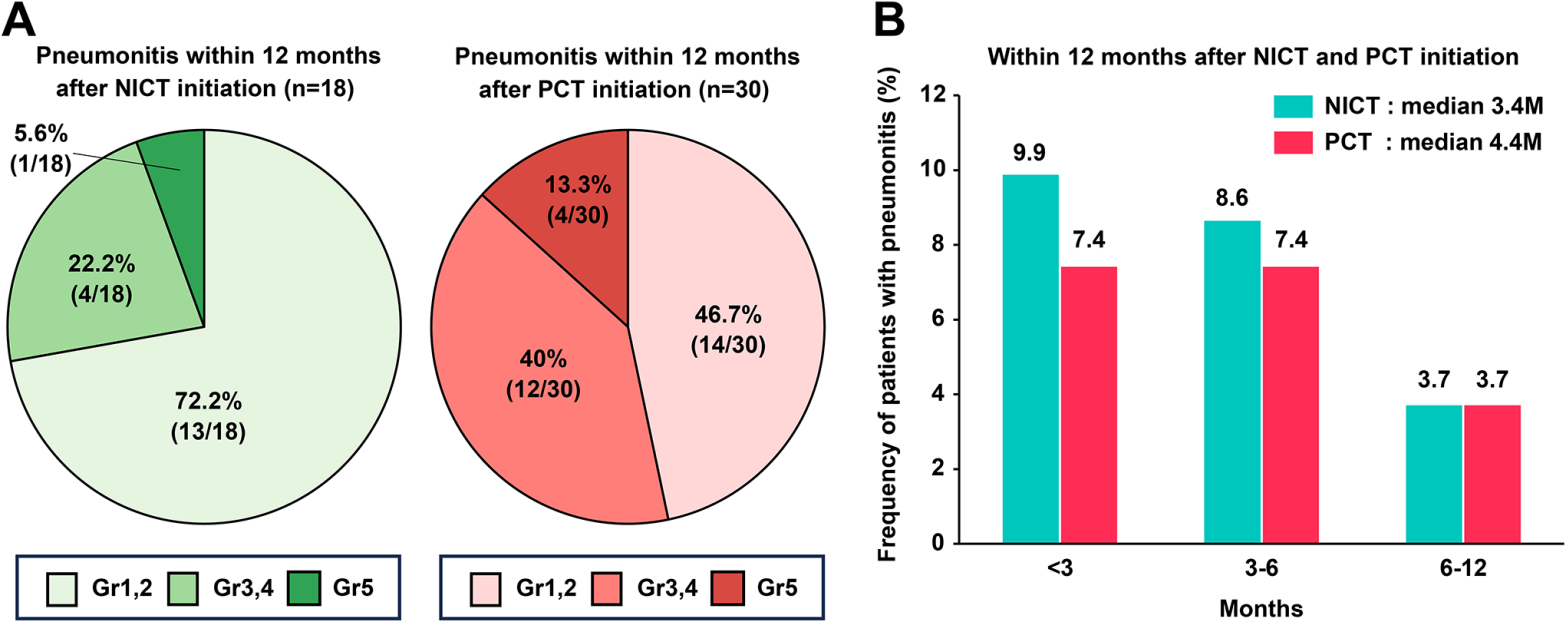
The frequency of pneumonitis development in patients was stratified according to grade within 12 months of NICT or PCT initiation (**A**); time from NICT and PCT initiation to the onset of any grade pneumonitis (**B**). Abbreviations: NICT, nivolumab plus ipilimumab with chemotherapy; PCT, pembrolizumab with chemotherapy

Among patients who developed pneumonitis, 18 (22.2%) and 30 (18.5%) developed any-grade pneumonitis within 12 months of combination therapy commencement in the NICT and PCT groups, respectively. The frequency of pneumonitis at each severity is shown in Fig. 3A. The median time to pneumonitis onset was 3.4 and 4.4 months in the NICT and PCT groups, respectively (Fig. 3B).

## Discussion

We evaluated the efficacy and safety of NICT and PCT in treatment-naïve patients with advanced NSCLC in a real-world setting using PSM. Patients who underwent NICT had significantly longer OS than those who underwent PCT, and a similar trend was observed in patients with PD-L1 TPS negative. Moreover, the safety profiles of NICT and PCT were comparable in terms of SAE, treatment discontinuation, and TRD rates. To the best of our knowledge, this is the first study to investigate the efficacy and safety of nivolumab plus ipilimumab and to directly compare NICT and PCT in real-world settings.

In this study, no significant differences were noted in short-term outcomes (i.e., TTD and PFS) between the NICT and PCT groups; however, a longer OS was observed in the NICT group. The addition of ipilimumab to anti-PD-1 antibody may have affected the longer survival by enhancing memory CD8 + T-cell function and decreasing regulatory T-cell via antibody-dependent cell-mediated cytotoxicity [15, 16]. In a network meta-analysis, O’Byrne et al. [17] demonstrated that the annual survival HR for nivolumab plus ipilimumab was the reverse of that for PCT after 12 months and remained superior to PCT thereafter. Moreover, the PFS-HR for PCT was equivalent or superior to that for nivolumab plus ipilimumab at 6 months. These results suggest NICT may still exhibit potential anti-tumor effects even after image-based disease progression by RECIST. In addition, the time from treatment discontinuation to disease progression was 2.3 months in the PCT group versus 5.2 months in the NICT group. Discontinuation due to mild toxicity was approximately 10% more common in the NICT group, which may have resulted in a longer immune response [18].

In subgroup analyses, similar trends were observed for subsets such as age ≥ 65 years, better PS, adenocarcinoma, and PD-L1 TPS negative. Currently, data on adding ipilimumab in elderly patients and those with poorer PS are limited. The Energy-GFPC 06-2015 study (the phase III trial of NI versus platinum-based chemotherapy in elderly and PS 2 patients) reported no benefits from the addition of ipilimumab for patients with PS 2 patients but highlighted a significant OS benefit in elderly patients [19]. NICT may provide similar advantages in elderly patients. In KEYNOTE-189/407 follow-up data, 5-year survival has gradually decreased as PD-L1 TPS decreased [20, 21]. In contrast, in the CheckMate-9LA trial, 3-year survival has remained constant, regardless of PD-L1 TPS [22]. These relative trends may have caused the difference in our OS data according to PD-L1 TPS; OS was significantly prolonged in the NICT group only when PD-L1 TPS < 1%. The reason for this difference in OS among different histology remains unknown as OS-HR is better in squamous cell carcinoma than in non-Squamous cell carcinoma in Checkmate-9LA (HR: 0.64, 95%CI: 0.48–0.86 vs. HR: 0.80, 95%CI: 0.65–0.98). However, the differences in genetic profile between the two groups may have affected OS (Table S2).

Investigating the safety of additional anti-CTLA-4 antibodies was essential for this study because the NIPPON study finally reported that 11 patients (7.4%) in the NICT group developed TRD [11]. The most common cause of death was pneumonitis in four cases, but the second most common cause was cytokine release syndrome (CRS). CRS has no specific diagnostic methods and may be among the SAEs of unknown cause. The cause of such a high TRD rate in the NIPPON study remains unclear. A subset analysis of Asian patients in CheckMate-9LA demonstrated that no TRD occurred regardless of SAEs in 21% of patients [23]. We also demonstrated that two (2.5%) patients in the NICT group died owing to SAEs, which was equivalent to the TRD rate (2%) in Checkmate-9LA. Thus, the TRD in the NIPPON study may be too high, not least because our TRD cases had no CRS and immunotherapy-induced CRS is rare worldwide [24, 25]. However, due to the existence of several reports, CRS should be monitored carefully during ICI combination therapy. In the NICT group, SAEs occurred in 28 (34.6%) patients, and skin and hepatobiliary toxicities and adrenal pituitary disorder occurred more frequently than in the PCT group. The CheckMate-9LA Asian subset analyses presented similar results, with skin toxicity being the most common SAE, followed by endocrine and hepatic disorders. Moreover, Gu et al. [26] reported that NICT results in a higher rate of dermatological and hepatic SAEs than nivolumab alone (risk ratios of 5.0 and 2.3, respectively). Hence, although it is not necessary to avoid using NICT owing to concerns regarding TRDs, these AEs should be noted when using NICT.

Patients who underwent PCT developed more severe pneumonitis than those who underwent NICT, and the TRDs in the PCT group included four cases of pneumonitis. In the KEYNOTE-189/407 trials, the frequency of severe pneumonitis was 2.7–3.2% [27]. Two real-world PCT datasets reported drug-related pneumonitis frequencies: Fujimoto et al. and Renaud et al. reported severe pneumonitis rates of 3.3% and 2.5% during their follow-up periods, respectively [28, 29], which were much lower than the rate observed in our study (9.9%). This is likely because a longer follow-up period was set in this study than in the other study (20.9 vs. 4.5 months). Finally, pneumonitis onset was similar in both groups, but the disease tended to be more severe in the PCT group. This indicates that patients receiving PCT should be more carefully monitored for pneumonitis than those receiving NICT during the disease course.

Despite the large multicenter cohort and novel findings, this study has some limitations, including its retrospective nature, the possibility of selection bias, and the relatively small sample size in the NICT group. In our cohort, driver gene mutations were not included as an adjustment factor; the PCT group had a much higher frequency of unknown mutations than the NICT group because comprehensive panel testing was not widely available at the time of PCT initiation. Moreover, almost all patients belonged to a single ethnic group (Japanese), which limits the generalizability of these results to other populations. Finally, PSM has some limitations despite being designed to reduce confounding biases [30]. First, confounding biases could not be eliminated for data that were not measured as covariates of propensity scores. In our study, we did not include metastatic sites as covariates but the other possible prognostic factors. Then, cases that were not matched were excluded from the analysis; thus, generalizability is limited. As we enrolled a larger number of patients who underwent PCT, we chose 1:2 nearest-neighbor matching to avoid substantial case exclusion during matching.

## Conclusions

Our study demonstrates differences in efficacy and safety in a real-world setting by comparing NICT with PCT through PSM. NICT provided longer OS benefits than PCT, and similar findings were observed for subsets such as the elderly, better PS, and PD-L1 TPS-negative. Safety profiles were almost comparable although there were unbalanced incidences of pneumonitis, skin and hepatic toxicities, and adrenal pituitary disorder. The real-world data suggest that NICT could be a favorable first-line treatment option compared with PCT for patients with advanced NSCLC with no EGFR and ALK genomic aberrations. Further investigation is warranted for long-term survival owing to the short follow-up period in this study.

### Electronic supplementary material

Below is the link to the electronic supplementary material.

Supplementary Material 1

## References

[1] Gandhi L, Rodríguez-Abreu D, Gadgeel S, Esteban E, Felip E, De Angelis F (2018). Pembrolizumab plus Chemotherapy in Metastatic Non-small-cell Lung Cancer. N Engl J Med.

[2] Paz-Ares L, Luft A, Vicente D, Tafreshi A, Gümüş M, Mazières J (2018). Pembrolizumab plus Chemotherapy for squamous non-small-cell Lung Cancer. N Engl J Med.

[3] Paz-Ares L, Ciuleanu T, Cobo M, Schenker M, Zurawski B, Menezes J (2021). First-line nivolumab plus ipilimumab combined with two cycles of chemotherapy in patients with non-small-cell Lung cancer (CheckMate 9LA): an international, randomised, open-label, phase 3 trial. Lancet Oncol.

[4] Socinski MA, Jotte RM, Cappuzzo F, Orlandi F, Stroyakovskiy D, Nogami N (2018). Atezolizumab for First-Line treatment of metastatic nonsquamous NSCLC. N Engl J Med.

[5] West H, McCleod M, Hussein M, Morabito A, Rittmeyer A, Conter HJ (2019). Atezolizumab in combination with carboplatin plus nab-paclitaxel chemotherapy compared with chemotherapy alone as first-line treatment for metastatic non-squamous non-small-cell Lung cancer (IMpower130): a multicentre, randomised, open-label, phase 3 trial. Lancet Oncol.

[6] Johnson ML, Cho BC, Luft A, Alatorre-Alexander J, Geater SL, Laktionov K (2023). Durvalumab with or without Tremelimumab in Combination with Chemotherapy as First-Line therapy for metastatic non-small-cell Lung Cancer: the Phase III POSEIDON Study. J Clin Oncol.

[7] Hellmann MD, Paz-Ares L, Bernabe Caro R, Zurawski B, Kim SW, Carcereny Costa E (2019). Nivolumab plus Ipilimumab in advanced non–small-cell Lung cancer. N Engl J Med.

[8] Liu L, Bai H, Wang C, Seery S, Wang Z, Duan J (2021). Efficacy and safety of First-Line Immunotherapy combinations for Advanced NSCLC: a systematic review and network Meta-analysis. J Thorac Oncol.

[9] Siciliano MA, Caridà G, Ciliberto D, d’Apolito M, Pelaia C, Caracciolo D (2022). Efficacy and safety of first-line checkpoint inhibitors-based treatments for non-oncogene-addicted non-small-cell Lung cancer: a systematic review and meta-analysis. ESMO Open.

[10] Shiraishi Y, Hakozaki T, Nomura S, Kataoka T, Tanaka K, Miura S (2022). A Multicenter, Randomized Phase III Study comparing platinum combination Chemotherapy Plus Pembrolizumab with Platinum Combination Chemotherapy Plus Nivolumab and Ipilimumab for Treatment-Naive Advanced Non-small Cell Lung Cancer without driver gene alterations: JCOG2007 (NIPPON Study). Clin Lung Cancer.

[11] Shiraishi Y, Sekino Y, Horinouchi H, Ohe Y, Okamoto I (2023) High incidence of cytokine-release syndrome in patients with advanced NSCLC treated with nivolumab plus ipilimumab. Ann Oncol 2(S0923–75342300827–X). 10.1016/j.annonc.2023.08.012 10.1016/j.annonc.2023.08.012 37666485

[12] Benedetto U, Head SJ, Angelini GD, Blackstone EH (2018). Statistical primer: propensity score matching and its alternatives. Eur J Cardiothorac Surg.

[13] Eisenhauer EA, Therasse P, Bogaerts J, Schwartz LH, Sargent D, Ford R (2009). New response evaluation criteria in solid tumours: revised RECIST guideline (version 1.1). Eur J Cancer.

[14] Common Terminology Criteria for Adverse Events (CTCAE). v.5.0 (2017) ; *Common Terminology Criteria for Adverse Events (CTCAE). v.5.0*. United States Department of Health and Human Services National Institutes of Health – National Cancer Institute, https://www.meddra.org/. Accessed May 31, 2023

[15] Chambers CA, Sullivan TJ, Truong T, Allison JP (1998) Secondary but not primary T cell responses are enhanced in CTLA-4-deficient CD8 + T cells. Eur J Immunol 28:3137–3143. 10.1002/(SICI)1521-4141(199810)28:10<3137::AID-IMMU3137>3.0.CO;2-X 10.1002/(SICI)1521-4141(199810)28:10<3137::AID-IMMU3137>3.0.CO;2-X 9808182

[16] Romano E, Kusio-Kobialka M, Foukas PG, Baumgaertner P, Meyer C, Ballabeni P (2015). Ipilimumab-dependent cell-mediated cytotoxicity of regulatory T cells ex vivo by nonclassical monocytes in Melanoma patients. Proc Natl Acad Sci U S A.

[17] O’Byrne K, Popoff E, Badin F, Lee A, Yuan Y, Lozano-Ortega G (2023). Long-term comparative efficacy and safety of nivolumab plus ipilimumab relative to other first-line therapies for advanced non-small-cell Lung cancer: a systematic literature review and network meta-analysis. Lung Cancer.

[18] Conde-Estevez D, Monge-Escartin I, Rios-Hoyo A, Monzonis X, Escheverria-Esnal D, Moliner L (2021). Prognostic factors and effect on survival of immune-related adverse events in patients with non-small-cell Lung cancer treated with immune checkpoint blokage. J Chemother.

[19] Lena H, Monnet I, Bylicki O, Audigier-Valette C, Falchero L, Vergnenegre A et al (2022) Randomized phase III study of nivolumab and ipilimumab versus carboplatin-based doublet in first-line treatment of PS 2 or elderly (≥ 70 years) patients with advanced non–small cell lung cancer. J Clin Oncol 40(16_suppl):9011–9011. 10.1200/JCO.2022.40.16_suppl.901

[20] Garassino MC, Gadgeel S, Speranza G, Felip E, Esteban E, Dómine M (2023). Pembrolizumab Plus Pemetrexed and Platinum in Nonsquamous Non-small-cell Lung Cancer: 5-Year outcomes from the phase 3 KEYNOTE-189 study. J Clin Oncol.

[21] Novello S, Kowalski DM, Luft A, Gümüş M, Vicente D, Mazières J (2023). Pembrolizumab plus chemotherapy in squamous non–small-cell Lung cancer: 5-year update of the phase III KEYNOTE-407 study. J Clin Oncol.

[22] Paz-Ares LG, Ciuleanu TE, Cobo M, Bennouna J, Schenker M, Cheng Y (2023). First-line nivolumab plus ipilimumab with chemotherapy versus chemotherapy alone for metastatic NSCLC in CheckMate 9LA: 3-year clinical update and outcomes in patients with brain metastases or select somatic mutations. J Thorac Oncol.

[23] John T, Sakai H, Ikeda S, Cheng Y, Kasahara K, Sato Y (2022). First-line nivolumab plus ipilimumab combined with two cycles of chemotherapy in advanced non-small cell Lung cancer: a subanalysis of Asian patients in CheckMate 9LA. Int J Clin Oncol.

[24] Kunimasa K, Inoue T, Matsueda K, Kawamura T, Tamiya M, Nishino K (2021). Cytokine Release Syndrome and Immune-related Pneumonitis Associated with Tumor Progression in a pulmonary pleomorphic carcinoma treated with Nivolumab plus Ipilimumab Treatment: a Case Report. JTO Clin Res Rep.

[25] Menakuru SR, Azeem Q, Priscu A, Khan I, Beirat A (2022). Stage 4 Cytokine Release Syndrome caused by the first dose of Nivolumab and Ipilimumab Combination Therapy in a patient with metastatic Melanoma successfully treated with methylprednisolone, Tocilizumab, and Etanercept. Case Rep Oncol.

[26] Gu J, Shi L, Jiang X, Wen J, Zheng X, Cai H (2022). Severe immune-related adverse events of immune checkpoint inhibitors for advanced non-small cell Lung cancer: a network meta-analysis of randomized clinical trials. Cancer Immunol Immunother.

[27] Paz-Ares L, Vicente D, Tafreshi A, Robinson A, Soto Parra H, Mazières J (2020). A randomized, placebo-controlled trial of Pembrolizumab Plus Chemotherapy in patients with metastatic squamous NSCLC: protocol-specified final analysis of KEYNOTE-407. J Thorac Oncol.

[28] Fujimoto D, Miura S, Yoshimura K, Wakuda K, Oya Y, Yokoyama T (2021). Pembrolizumab plus chemotherapy-induced pneumonitis in chemo-naïve patients with non-squamous non-small cell Lung cancer: a multicentre, retrospective cohort study. Eur J Cancer.

[29] Renaud E, Ricordel C, Corre R, Leveiller G, Gadby F, Babey H (2023). Pembrolizumab plus pemetrexed-carboplatin combination in first-line treatment of advanced non-squamous non-small cell Lung cancer: a multicenter real-life study (CAP29). Transl Lung Cancer Res.

[30] Okoli GN, Sanders RD, Myles P (2014). Demystifying propensity scores. Br J Anaesth.

