# Supplementary material for: Real-world outcomes of nivolumab plus ipilimumab and pembrolizumab with platinum-based chemotherapy in advanced non-small cell lung cancer: a multicenter retrospective comparative study
Source: Cancer Immunol Immunother. 2024 Jan 4;73(1):4. doi: 10.1007/s00262-023-03583-4 (PMC10766714; doi:10.1007/s00262-023-03583-4)
Supplement: Supplementary file 1 — Supplementary Material 1 [file 262_2023_3583_MOESM1_ESM.docx]

**Real-world outcomes of nivolumab plus ipilimumab and pembrolizumab with platinum-based chemotherapy in advanced non-small cell lung cancer: A multicenter retrospective comparative study**

**Fig. S1** Patient flow chart.

**Fig. S2**Subgroup analyses for time to treatment discontinuation in the NICT and PCT groups after a propensity score matching method according to age (A1: <65 years, A2: ≥65 years), ECOG-PS (B1: PS 0–1, B2: PS 2–4), histology (C1: adenocarcinoma, C2: squamous cell carcinoma), and PD-L1 TPS (D1: TPS ≥50%, D2: TPS 1–49%, D3: TPS <1%). Abbreviations: NICT, nivolumab plus ipilimumab with chemotherapy; PCT, pembrolizumab with chemotherapy; mTTD, median time to treatment discontinuation; HR, hazard ratio; ECOG-PS, Eastern Cooperative Oncology Group Performance Status; PD-L1 TPS, programmed cell death ligand-1 tumor proportion score.


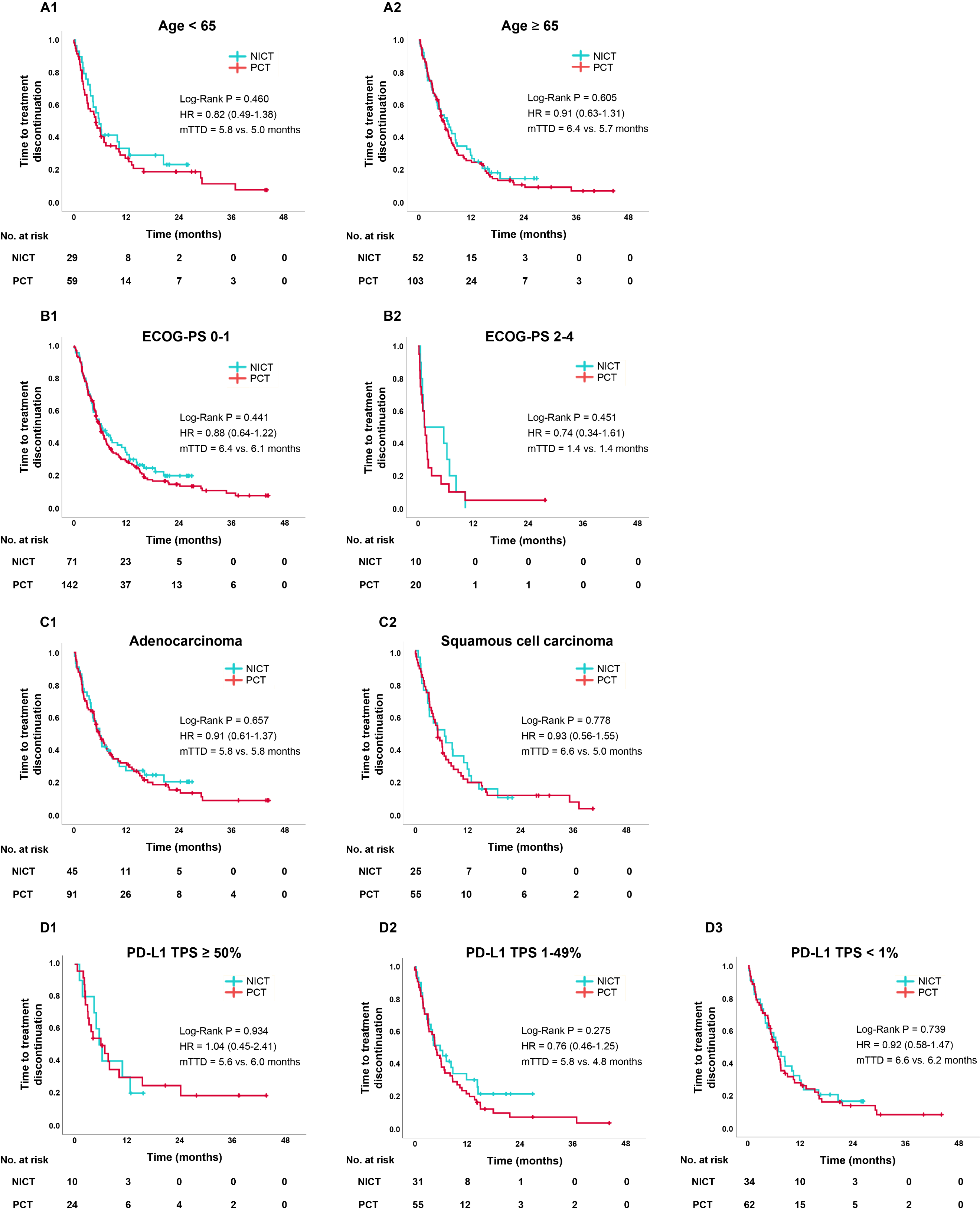


**Fig. S3**Subgroup analyses for progression-free survival in the NICT and PCT groups after a propensity score matching method according to age (A1: <65 years, A2: ≥65 years), ECOG-PS (B1: PS 0–1, B2: PS 2–4), histology (C1: adenocarcinoma, C2: squamous cell carcinoma), and PD-L1 TPS (D1: TPS ≥50%, D2: TPS 1–49%, D3: TPS <1%). Abbreviations: NICT, nivolumab plus ipilimumab with chemotherapy; PCT, pembrolizumab with chemotherapy; mPFS, median progression free survival; HR, hazard ratio; ECOG-PS, Eastern Cooperative Oncology Group Performance Status; PD-L1 TPS, programmed cell death ligand-1 tumor proportion score.


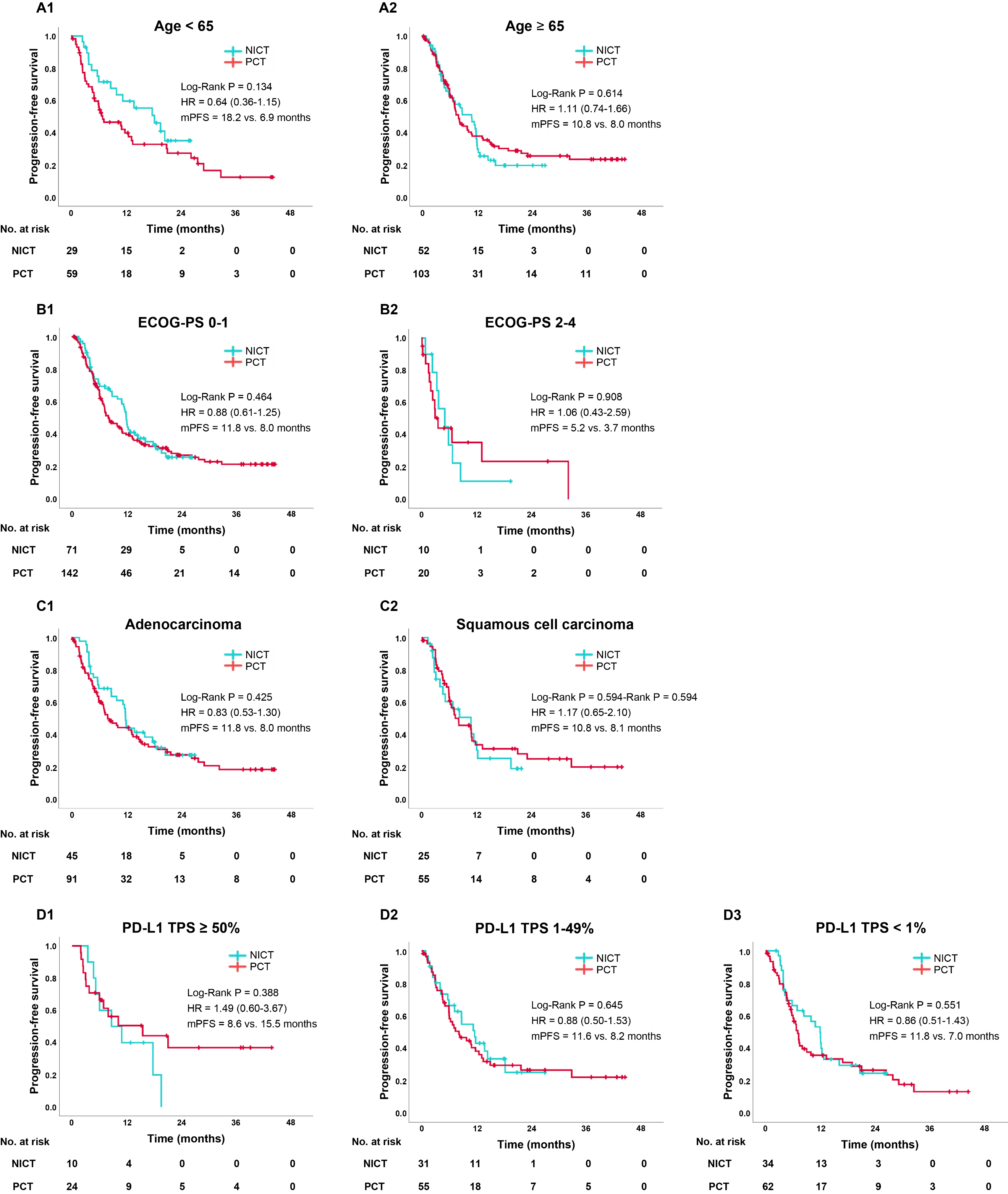


**Table S1** Treatment characteristics for matched patients.

|  | NICT  (n=81) | PCT  (n=162) | P value |
| --- | --- | --- | --- |
| Chemotherapy regimen, n (%) |  |  | 0.674 |
| Cisplatin or carboplatin/pemetrexed | 49 (60.5) | 103 (63.6) |  |
| Carboplatin/paclitaxel or nab-paclitaxel | 32 (39.5) | 59 (36.4) |  |
| Number of treatment lines, n (%) |  |  | 0.102 |
| 1 | 37 (45.7) | 92 (56.8) |  |
| ≥2 | 44 (54.3) | 70 (43.2) |  |
| Tumor response, n (%) |  |  |  |
| Complete response | 4 (4.9) | 4 (2.5) |  |
| Partial response | 54 (66.7) | 90 (55.6) |  |
| Stable disease | 13 (16.0) | 47 (29.0) |  |
| Progression disease | 8 (9.9) | 17 (10.5) |  |
| Not evaluated | 2 (2.5) | 4 (2.5) |  |
| Objective response rate, n (%) | 58 (71.6) | 94 (58.0) | 0.039 |
| Disease control rate, n (%) | 71 (87.7) | 141 (87.0) | 0.892 |

Abbreviations: NICT, nivolumab plus ipilimumab with chemotherapy; PCT, pembrolizumab with chemotherapy.

**Table S2** Driver gene mutation.

|  | Patients adjusted after PSM (n=243) | |  |
| --- | --- | --- | --- |
|  | NICT  (n=81) | PCT  (n=162) | P value |
| Unknown | 7 (8.6) | 88 (54.3) | <0.001 |
| Negative | 57 (70.4) | 59 (36.4) | <0.001 |
| Positive | 17 (21.0) | 15 (9.3) | 0.011 |
| AKT1 mutation | 1 (1.2) | 0 (0) |  |
| BRAF mutation | 2 (2.5) | 0 (0) |  |
| CTNNB1 mutation | 1 (1.2) | 0 (0) |  |
| ERBB2 mutation | 0 (0) | 1 (0.6) |  |
| EGFR minor mutation | 0 (0) | 4 (2.5) |  |
| FGFR mutation | 1 (1.2) | 0 (0) |  |
| KRAS mutation | 11 (13.6) | 5 (3.1) |  |
| MET mutation | 0 (0) | 2 (1.2) |  |
| PIK3CA mutation | 1 (1.2) | 1 (0.6) |  |
| RET mutation | 0 (0) | 2 (1.2) |  |
| NA | 1 (1.2) | 0 (0) |  |

Abbreviations: PSM, propensity score matching; NICT, nivolumab plus ipilimumab with chemotherapy; PCT, pembrolizumab with chemotherapy.
